# Humanized-Single Domain Antibodies (VH/V_H_H) that Bound Specifically to *Naja kaouthia* Phospholipase A2 and Neutralized the Enzymatic Activity

**DOI:** 10.3390/toxins4070554

**Published:** 2012-07-19

**Authors:** Charnwit Chavanayarn, Jeeraphong Thanongsaksrikul, Kanyarat Thueng-in, Kunan Bangphoomi, Nitat Sookrung, Wanpen Chaicumpa

**Affiliations:** 1 Graduate Program in Immunology, Faculty of Medicine Siriraj Hospital, Mahidol University, Bangkok 10700, Thailand; Email: sandwashed@hotmail.com; 2 Laboratory for Research and Technology Development, Department of Parasitology, Faculty of Medicine Siriraj Hospital, Mahidol University, Bangkok 10700, Thailand; Email: gskmu@hotmail.com (J.T.); mam_mt41@hotmail.com (K.T.); 3 Department of Biochemistry, Kasetsart University, Bangkok 10900, Thailand; Email: kunan_b@hotmail.com; 4 Department of Research and Development, Faculty of Medicine Siriraj Hospital, Mahidol University, Bangkok 10700, Thailand; Email: nitat.soo@mahidol.ac.th

**Keywords:** snake bite, snake venom, phospholipase A2 (PLA_2_), single domain antibody (SdAb), VH/V_H_H, homology modeling, molecular docking

## Abstract

*Naja kaouthia* (monocled cobra) venom contains many isoforms of secreted phospholipase A2 (sPLA_2_). The PLA_2_ exerts several pharmacologic and toxic effects in the snake bitten subject, dependent or independent on the enzymatic activity. *N. kaouthia* venom appeared in two protein profiles, P3 and P5, after fractionating the venom by ion exchange column chromatography. In this study, phage clones displaying humanized-camel single domain antibodies (VH/V_H_H) that bound specifically to the P3 and P5 were selected from a humanized-camel VH/V_H_H phage display library. Two phagemid transfected *E. coli* clones (P3-1 and P3-3) produced humanized-V_H_H, while another clone (P3-7) produced humanized-VH. At the optimal venom:antibody ratio, the VH/V_H_H purified from the *E. coli* homogenates neutralized PLA_2_ enzyme activity comparable to the horse immune serum against the *N. kaouthia* holo-venom. Homology modeling and molecular docking revealed that the VH/V_H_H covered the areas around the PLA_2_ catalytic groove and inserted their Complementarity Determining Regions (CDRs) into the enzymatic cleft. It is envisaged that the VH/V_H_H would ameliorate/abrogate the principal toxicity of the venom PLA_2_ (membrane phospholipid catabolism leading to cellular and subcellular membrane damage which consequently causes hemolysis, hemorrhage, and dermo-/myo-necrosis), if they were used for passive immunotherapy of the cobra bitten victim. The speculation needs further investigations.

## 1. Introduction

Venoms of poisonous snakes contain several isoforms of secreted phospholipase A2 (sPLA_2_) [1,2,3]. The principal role of the snake venom PLA_2_ is for digesting the prey. Nevertheless, PLA_2_ is regarded as one of the major and the most toxic components of the venoms causing several pharmacologic effects and toxicities, either dependent or independent of the catalytic activity, to the snake bitten subjects [4,5,6]. This enzyme has phosphatidylcholine 2-acid hydrolase activity which specifically hydrolyses the ester bonds at position 2 of 1,2 diacyl-*sn*-3-phosphoglycerides to produce free fatty acid and lysophospholipids [6]. PLA_2_ digests cell membrane phospholipids yielding arachidonic acid which is metabolized further to form pro-inflammatory eicosanoids, including prostaglandins (cyclooxygenase metabolic pathway) and leukotrienes and platelet activating factors (PAF) (lypooxygenase metabolic pathway) [7]. Besides the inflammation, particularly tissue edema and pain caused by the eicosanoids, the PLA_2_ causes, as a consequence of the cellular and subcellular (mitochondrial) membrane damage, lysis of erythrocytes, hemorrhage and dermo-/myo-necrosis [4,8,9]. The poisonous snake victims suffer also blood coagulopathy due to the PLA_2 _mediated inhibition of platelet aggregation and fibrinogenolysis [10,11,12,13], cardiotoxicity [4,14] as well as pre- and post-synaptic neurotoxicity [15]. 

Passive administration of antivenom derived from animal immunized with appropriate holo-snake venom is the only specific treatment for snake bite [4]. Nevertheless, the effectiveness of the antivenom therapy in neutralizing the local dermonecrosis, especially from the cobra bites, is unappreciated [4,15]. Moreover, the animal foreign proteins induce frequently adverse side effects including early phase allergic reactions which may be as severe as anaphylaxis and later the detrimental serum sickness. Several natural inhibitors of snake PLA_2_ activities have been searched for adjunctive use in attenuation of the enzyme mediated toxicities [8,15]. These include protein inhibitors from endogenous blood of several snake species and mammalian blood, as well as inhibitors from medicinal plants [8]. The mechanism of the natural inhibitors in abolishing/ameliolating the sPLA_2_ activities was proposed [8]; including inhibition of p38 MAPK phosphorylation which slow-down the transcription factors specific for transcription of various matrix metalloproteinases and inflammatory cytokines. 

Camelids including old world camel (one hump) and llama contained in their sera, not only the conventional 2H2L IgG immunoglobulins, but also atypical antibodies which the molecules devoid of L chains and consist of only H chains in homodimeric form. These antibodies are called heavy chain antibodies (HCAb). H chain of the HCAb lacks CH1 domain and the hinge region is exceptionally long. Antigen binding domain of the HCAb is called V_H_H domain in order to distinguish from the heavy chain variable domain (VH) of the conventional four chain antibodies. VH/V_H_H (alternatively called single domain antibodies, SdAb) are small (15–20 kDa); thus, they have high tissue penetrating ability and rapid bio-distribution. V_H_H has been shown to be potent enzyme inhibitor [16,17,18,19,20]. Recently, a humanized-camel VH/V_H_H phage display library was constructed [19]. Humanized-camel VH/V_H_H sequences from the phage library showed high sequence identity to the human VH sequences indicating their negligible immunogenicity to the human immune system if they were used for passive immunotherapy. V_H_H specific to botulinum neurotoxin derived from this library neutralized readily the zinc metalloproteinase activity of the neurotoxin light chain by inserting the CDR3 domain directly into the toxin catalytic groove [19]. This enzyme inhibitory mechanism cannot be achieved from the large sized antibody molecules such as intact IgG (150 kDa). Therefore in this communication, humanized-camel SdAb that bound specifically with PLA_2_ of *Naja kaouthia* (monocled cobra) which is a predominant snake species causing high hospitalized cases and relatively high mortality among the bitten victims in Thailand, were produced and tested for neutralization of enzymatic activity of the PLA_2_. 

## 2. Materials and Methods

### 2.1. *N. kaouthia* Venom and Horse Immune Serum against *N. kaouthia* Venom

*N. kaouthia* venom and horse immune serum to *N. kaouthia* venom were obtained from the Queen Saovabha Memorial Institute, Thai Red Cross, Bangkok, Thailand. Universal precaution was followed when handling the venom. The venom was dissolved in small volume of sterile distilled water and the protein content was measured using Bradford reagent. The venom solution was fractionated by cation exchange column chromatography [21,22]. After the cellulose matrix was well equilibrated with 0.09 M ammonim acetate, pH 6.5, the venom solution was loaded onto the column and the column flow-through fluids were collected in three ml fractions; the bound proteins were eluted with a gradient of 0.14 to 1.2 M ammonium acetate, pH 6.5 and also collected in three ml fractions [22]. OD_280nm_ of each fraction was monitored. The protein peaks 3 and 5 (P3 and P5, respectively) which had been shown by LC-MS/MS to be PLA_2_ of the *N. kaouthia* [22] were dialysed against distilled water, concentrated, and the protein contents were measured. 

### 2.2. Humanized-Camel VH/V_H_H Phage Display Library

The humanized-camel VH/V_H_H phage display library used in this study was constructed previously [19]. Briefly, cDNA was prepared from mRNA of lymphocytes of a naïve camel, *Camelus dromedarius,* and used as template for amplification of VH and V_H_H by PCR. The oligonucleotide primers used for the PCR, however, were human degenerate primers designed from all families of human immunoglobulin *vh* and *jh* sequences [22]. Thus, the human primers directed amplification of only human-like camel *vh/vhh* (humanized-) sequences. The humanized-*vh/vhh* sequences were ligated with pCANTAB5E phagemid DNA and the recombinant phagemids were used to transfect appropriate competent *E. coli*. After growing the recombinant phagemid transformed *E. coli* in the presence of helper phage (M13KO7), complete phage particles which displayed humanized-VH/V_H_H as a fusion protein on the phage coat and also carried the respective *vh/vhh* in the phage genome could be obtained from the *E. coli* culture supernatant. They were used in the phage bio-panning below.

### 2.3. Phage Bio-Panning for Selecting Phage Clones that Displayed P3- and P5-Bound VH/V_H_H from the Phage Library

The P3 and P5 PLA_2_ purified from the *N. kaouthia* venom were used separately as antigens in the single round-phage bio-panning which was done as described previously [19,22]. One microgram of P3/P5 protein was immobilized on the surface of separate wells of microtiter plate (Costar, Corning, USA). The humanized VH/V_H_H phage display library was added into the antigen coated wells and kept at 25 °C for 1 h. The unbound phage particles were removed by extensive washing with a washing buffer. Bound phage particles were immediately supplemented with log-phase grown HB2151 *E. coli* bacteria. The phagemid transformed HB2151 *E. coli* preparations were spread on LB-AG (LB-A containing 2% glucose) agar plates and the plates were incubated at 37 °C overnight. Colonies grown on plates were randomly picked and screened for the recombinant *vh/v_h_h*-phagemids by PCR using phagemid specific primers, *R1* and *R2*. The *E. coli* transformants positive for the recombinant *vh/v_h_h*-phagemid vectors were further screened for their ability to express soluble VH/V_H_H by indirect ELISA. 

### 2.4. Screening of the Transformed *E. coli* Clones that Could Express VH/V_H_H

The *E. coli* clones positive for *vh/vhh* sequences were grown individually in LB-A broth containing 0.5 mM IPTG for 5 h. The bacterial cells were collected and subjected to sonication and centrifugation. Individual bacterial lysates were screened for the presence of VH/V_H_H by Western blot analysis (WB). Each lysate was electrophoretically separated in 12% polyacrilamide gel and the separated components were blotted onto nitrocellulose membrane (NC). After blocking the unoccupied sites on the NC with unrelated protein, the NC blot was probed with rabbit anti-E Tag antibody (Abcam^®^, Cambridge, UK). The VH/V_H_H bound-rabbit anti-E tag was revealed by using goat anti-rabbit immunoglobulin-alkaline phosphatase (AP) conjugate (Southern Biotech) and BCIP/NBT chromogenic substrate. The VH/V_H_H contained in each lysate was standardized spectrophotometrically based on the band intensities in the WB. The standardized VH/V_H_H in the bacterial lysates were subjected to indirect ELISA and Western blot analysis for determining their specific binding to the P3 and P5 proteins, or both. 

### 2.5. Determination of Specific Binding of the VH/V_H_H to the P3 and P5 PLA_2_

For indirect ELISA, P3 and P5 were used for coating separate ELISA wells. Wells coated with bovine serum albumin (BSA), lysate of original HB2151 *E. coli* and PBS only were included as antigen control, background, and blank, respectively. After being incubated and washed, wells were added appropriately with individual *E. coli* lysates containing standardized VH/V_H_H. Rabbit anti-E tag, goat anti-rabbit immunoglobulin-horseradish peroxidase and substrate were sequentially added with washing between each step. OD_405nm_ of the contents in wells coated with *E. coli* lysates and BSA were determined against the blank. VH/V_H_H in *E. coli* lysates that revealed OD_405nm_ two times higher than the same lysates added to BSA coated wells were regarded as positive binding of the VH/V_H_H to the P3/P5, or both. 

For Western blot analysis, the SDS-PAGE separated P3 and P5 were electrotransblotted onto separate NC. After blocking, each NC blot was cut vertically into strips and immersed appropriately into standardized VH/V_H_H positive *E. coli* lysates. The VH/V_H_H that bound to P3/P5 on NC strip was revealed by using rabbit anti-E tag, goat anti-rabbit immunoglobulin-AP conjugate and enzyme chromogenic substrate, respectively. 

### 2.6. Determination of the Restriction Fragment Length Polymorphism (RFLP) of the *vh/v_h_h* Sequences

RFLP of DNA sequences coding for the VH/V_H_H in individual transformed HB2151 *E. coli* clones were determined after digesting with *Mva*I restriction endonuclease and resolved by 14% polyacrylamide gel electrophoresis followed by ethidium bromide staining [19].

### 2.7. Amino Acid Sequences, Immunoglobulin Frameworks (FRs) and Complementarity Determining Regions (CDRs) of the VH/V_H_H

The *vh/v_h_h* cDNA insert in the recombinant pCANTAB5E vector from each phagemid-transformed HB2151 *E. coli* clone was sequenced and deduced into amino acid sequence. The VH/V_H_H sequences were compared by ClustalW. The immunoglobulin frameworks (FRs) and complementarity determining regions (CRDs) of each VH/V_H_H were predicted by using the International ImMunoGeneTics (IMGT) information system [23].

### 2.8. Large Scale Production and Purification of the VH/V_H_H

The VH/V_H_H were produced in large scale by subcloning the *vh/v_h_h* inserts from pCANTAB5E to the modified pET23b^+^ vector backbone as described previously [24]. The recombinant *vh/v_h_h*-pET23b^+^vectors were introduced into BL21 (DE3) *E. coli*. The hexahistidine-tagged VH/V_H_H fusion proteins were produced from selected transformed bacteria and purified by using Ni-NTA agarose beads. The purified VH/V_H_H antibodies were tested for their ability to inhibit *N. kaouthia* PLA_2_ enzymatic activity in comparison with the horse anti-*N. kaouthia* venom.

### 2.9. PLA2 Enzymatic Assay and Neutralization of the Enzymatic Activity by Humanized-VH/V_H_H and Horse Anti-*N. kaouthia* Venom

The P3 and P5 enzymatic activity in hydrolyzing phosphatidylcholine substrate was determined by using secretory PLA_2_ assay kit (Cayman Chemical Company, MI, USA) according to manufacturer’s instruction. To set up the assay, 10 µL of DTNB, 10 µL P3/P5 (containing various amounts: 50, 100 and 150 ng) and 5 µL of assay buffer were mixed in each well of an ELISA plate (Corning). To initiate the reaction, the mixture was added to 200 µL of substrate solution (diheptanoyl thiol-phosphatidylcholine). Mixture added to deionized water and bee venom PLA_2_ instead of P3/P5 served as blank and positive enzyme control, respectively. A thio-ester bond of phosphatidylcholine was hydrolyzed by PLA_2_ and the free thiol group was detected by adding 5,5’-dithiol-*bis*-(2-nitrobenzoic Acid) (DTNB). OD_405nm_ of the reaction mixtures were determined spectrophotometrically at 1-min intervals. Activity of PLA_2_ (µmol/min/mL) was calculated from duplicate wells by formula: 

ΔOD_405nm_/min = [(OD_405nm_ at time-2) − (OD_405nm_ at time-1)] ÷ [time-2 (min) − time-1 (min)]

PLA_2_ activity = (ΔOD_405nm_/min ÷ 10.66 mM^−1^) × (0.225 mL ÷ 0.01 mL) × sample dilution

For the antibody mediated PLA_2_ neutralization assay, P3 and P5 was pre-incubated with purified VH/V_H_H for 1 h at ambient temperature. The P3/P5 pre-incubated with 10 µL of 1:1000 horse anti-*N. kaouthia* venom served as positive inhibition control. The reaction was initiated by adding DTNB and substrate solution. The OD_405nm _was measured and PLA_2_ activity was calculated as above. The experiments were repeated two times with high reproducibility.

### 2.10. Homology Modeling and Molecular Docking for Determination of the Interface Binding between the VH/V_H_H and the P3 and P5 PLA_2_

The structures of VH/V_H_H and PLA_2_ were modeled from their templates derived from BLAST search analysis. Homology modeling technique was used for constructing the antibody and the enzyme models. Steric hindrance of each modeled structure was determined by using RAMACHANDRAN plot. The structures of the VH/V_H_H and phospholipase A2 were subjected to molecular docking. All experiments were performed by using program Discovery studio 2.5. The enzyme and the antibodies were set as receptor and ligands, respectively. The docked poses from ZDOCK were subjected to structural refinement by using RDOCK program. 

## 3. Results

### 3.1. PLA_2_ Fractions from *N. kaouthia* Venom

After loading the *N. kaouthia* holo-venom onto the cationic exchange chromatography, 5 protein profiles (P1-P5) were washed through the column by using 0.09 M ammonium acetate, pH 6.5 and 6 profiles (P6-P11) of the column bound proteins were eluted out with a gradient solution of 0.14 to 1.2 M ammonium acetate [22]. Purity of the P3 and P5 profiles of the column flow through were determined by SDS-PAGE (Supplemental Figure 1). The proteins were identified as PLA_2_ by LC-MS/MS (Supplemental Figure 2). They were dialyzed and concentrated for further use. The P3 and P5 constituted 3.35 and 2.35% of the total venom proteins, respectively. 

### 3.2. Enzymatic Activity of the P3 and P5 from *N. kaouthia* Venom

By using the sPLA_2_ assay kit, both P3 and P5 preparations were found to have PLA_2_ catalytic activity. The optimal amounts of the proteins that gave appropriate enzymatic kinetics were 50 and 100 ng, respectively (data not shown). From duplicate experiments, the calculated PLA_2_ catalytic activity of the P3 and the P5 were 0.47 and 0.13 (µmol/min/mL), respectively.

### 3.3. Selection of Phage Clones that Displayed P3/P5-bound *VH/V_H_H* and Characterization of the VH/V_H_H

Both P3 and P5 proteins were used separately as antigens in the single round phage bio-panning. The recombinant pCANTAB5E-transformed HB2151 *E. coli* colonies appeared on the overnight plate, were screened for the presence of the *vh/v_h_h*. It was found that 21 of 30 clones from the P3-panning and 14 of 18 clones from the P5-panning were positive for the antibody gene sequences (~600 bp) by PCR and among them 17 and 12 clones, respectively, could express VH/V_H_H (*Mr* ~15–25 kDa) as determined Western blot analysis (representatives are shown in Supplemental Figure 3). 

**Figure 1:**
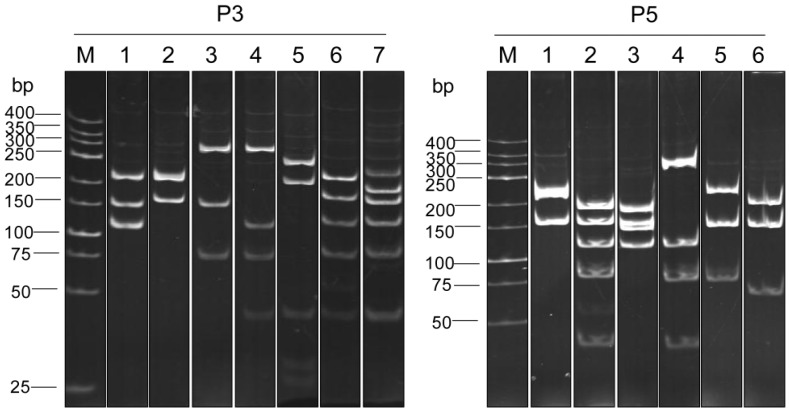
*Mva*I restriction RFLP patterns of the *vh/v_h_h* sequences of the clones that bound to the P3 and the P5, respectively. The P3 derived clones revealed 7 different DNA banding patterns while the P5 derived clones showed 6 different patterns.

RFLP of the *vh/v_h_h* sequences of the 17 and 12 clones were studied using *Mva*I restriction nuclease. The P3 derived clones revealed 7 different DNA banding patterns while the P5 derived clones showed 6 different RFLP patterns (Figure 1). 

**Figure 2:**
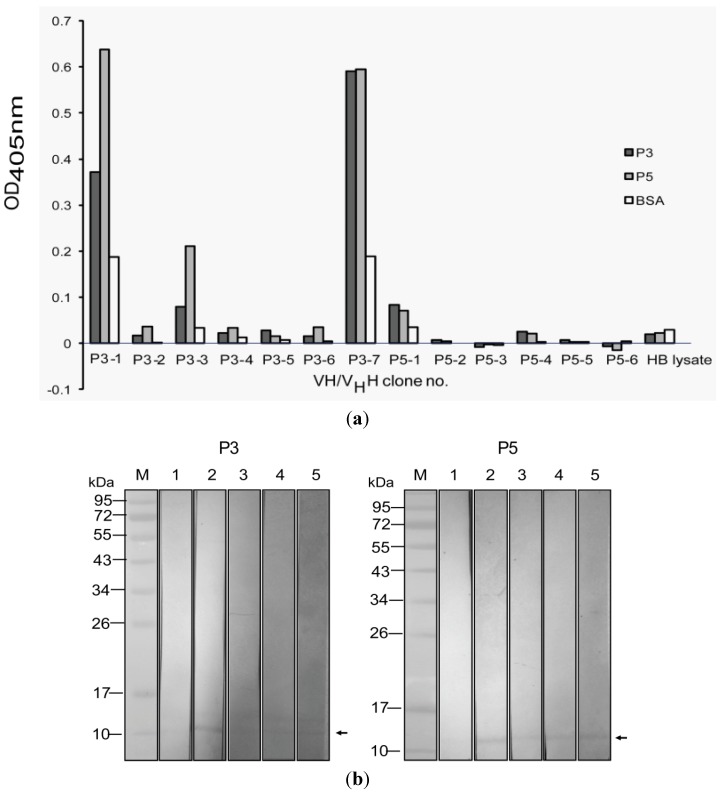
Binding specificity of VH/V_H_H in lysates of the selected *vh/v_h_h*-phagemid transformed HB2151 *E. coli* clones. (**a**) VH/V_H_H in lysates of 13 HB2151 *E. coli* clones were tested for binding to the P3 and the P5 by indirect ELISA; lysates of three clones, P3-1, P3-3 and P3-7, gave OD_405nm_ to the immobilized P3 and P5 two times higher than to BSA control. HB, Lysate of normal HB2151 *E. coli* used as negative VH/V_H_H control. (**b**) Western blot analysis for confirmation of the binding of the VH/V_H_H of the three ELISA positive *E. coli* clones to SDS-PAGE separated P3 and P5. VH/V_H_H of all clones bound to both proteins (lanes 3–5 of both panels) (arrows). Lanes M, Pre-stained protein marker; lanes 1, SDS-PAGE separated-P3/P5 probed with normal HB2151 *E. coli* lysate (negative control); lanes 2, SDS-PAGE separated-P3/P5 probed with horse anti-venom (1:1000) (positive control).

VH/V_H_H of representative clone of each pattern, designated clones P3-1 to P3-7 and P5-1 to P5-6 were tested for specific binding to the P3 and the P5 by indirect ELISA and Western blot analysis. It was found that VH/V_H_H in the lysates of clones P3-1, P3-3 and P3-7 bound to the P3 as well as the P5 by the indirect ELISA (Figure 2a). They also bound to the SDS-PAGE separated purified P3 and P5 PLA2 in the Western blot analysis (Figure 2b). None of the VH/V_H_H of clones P5-1 to P5-6 bound to P3 and/or P5 by both assays.

**Figure 3:**
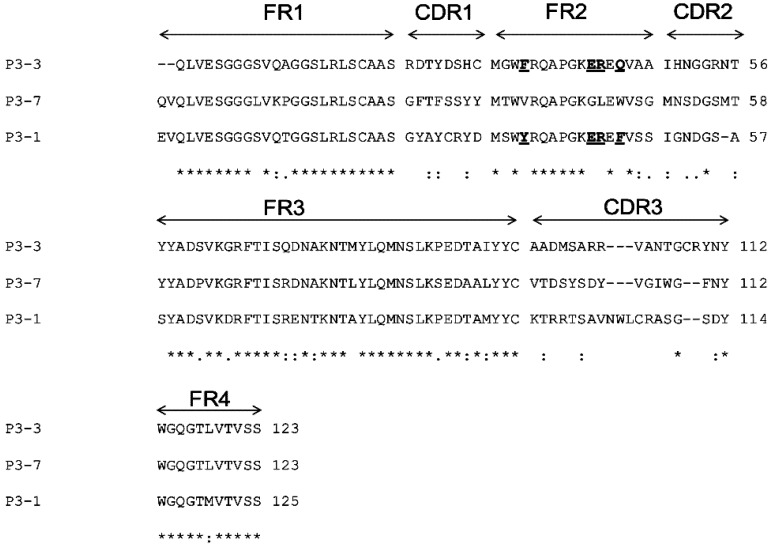
Multiple alignment of amino acid sequences for determining immunoglobulin frameworks (FRs) and complementarity determining regions (CRDs) of the VH/V_H_H sequences by using the International Immunogenetics Information System sever. Clones P3-1 and P3-3 showed the amino acid tetrad hallmark of V_H_H in the FR2 (underlined bold letters); clone P3-7 was conventional VH. Asterisk indicates identical amino acids; colon indicates conserved amino acid substitution; and dot indicates semi-conserved amino acid substitution.

Multiple alignments revealed that the amino acid sequences of VH/V_H_H of clones P3-1, P3-3 and P3-7 were different especially at the CDR domains (Figure 3). The deduced amino acid sequences of clones P3-1 and P3-3 had the characteristic amino acid tetrad of V_H_H in the immunoglobulin framework-2 (FR2); they were designated V_H_H-P3-1 and V_H_H-P3-3; the clone P3-7 had conventional VH features, designated VH-P3-7 [19]. 

All of the three humanized-camel VH/V_H_H showed high homology with human VH sequences (Table 1). 

**Table 1:** Percent amino acid identity of the humanized-camel VH/V_H_H sequences with the closest human V region frameworks [23].

VH/V_H_H clone number	Closest human V region	Percent amino acid identity with human FRs		
		FR1	FR2	FR3
V_H_H-P3-1	Z27054 IGHV3-66*02	92.0	70.6	78.9
V_H_H-P3-3	Z27054 IGHV3-66*02	84.0	58.8	84.2
VH-P3-7	HM855939	92.0	88.2	84.2

* Indicates the allele polymorphism.

### 3.4. VH/V_H_H-Mediated Inhibition of PLA_2_ Enzymatic Activity

**Figure 4:**
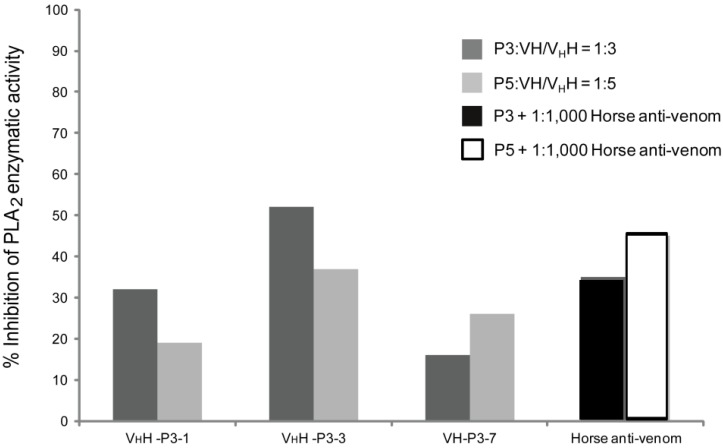
Results of VH/V_H_H- mediated inhibition of the P3 and the P5 enzymatic activity. P3 and P5 were mixed with V_H_H-P3-1, V_H_H-P3-3 and VH-P3-7 at molecular ratios 1:3 and 1:5 (antibody:enzyme), respectively. Horse anti-*N. kaouthia* venom diluted 1:1000 mixed with the P3/P5 was used as positive inhibition control. It was found that the V_H_H-P3-1, V_H_H-P3-3 and VH-P3-7 could inhibit the P3 enzyme (homologous system) by 32, 52 and 16%, respectively; the antibodies inhibited P5 enzyme (heterologous system) by 19, 37 and 26%, respectively. The horse immune serum at dilution 1:1000 inhibited the P3 and the P5 PLA_2_ by 35 and 45%, respectively.

The purified V_H_H-P3-1, V_H_H-P3-3 and VH-P3-7 when mixed with the P3 and the P5 at the optimal enzyme:antibody ratios 1:3 and 1:5, respectively, could inhibit activity of the enzymes by 32, 52 and 16% and 19, 37 and 26%, respectively (Figure 4). The horse anti-cobra venom (1:1000) inhibited catalytic activity of the P3 and the P5 PLA_2_ by 35 and 45%, respectively. 

### 3.5. Interface Binding of the VH/V_H_H and the PLA_2_

BLAST search analysis revealed that the amino acid sequences of VH/V_H_H of clones no. V_H_H-P3-1, V_H_H-P3-3, and VH-P3-7 had the highest sequence identity with PDB entries: 1VHP, 1MVF, and 2GCY, respectively. The PDB entry 1POA has the highest sequence identity with sPLA_2_. After homology modeling, the qualities of the modeled structures were assessed by RAMACHANDRAN plot. Percentages of amino acids in allowed regions of the VH/V_H_H clones no. P3-1, P3-3, and P3-7 were 99.1%, 98.2%, and 99.1% respectively (Supplemental Figure 4a). For modeled P3 and P5 PLA_2_, all amino acids (100%) of both structures were in the allowed regions (Supplemental Figure 4b). 

**Figure 5:**
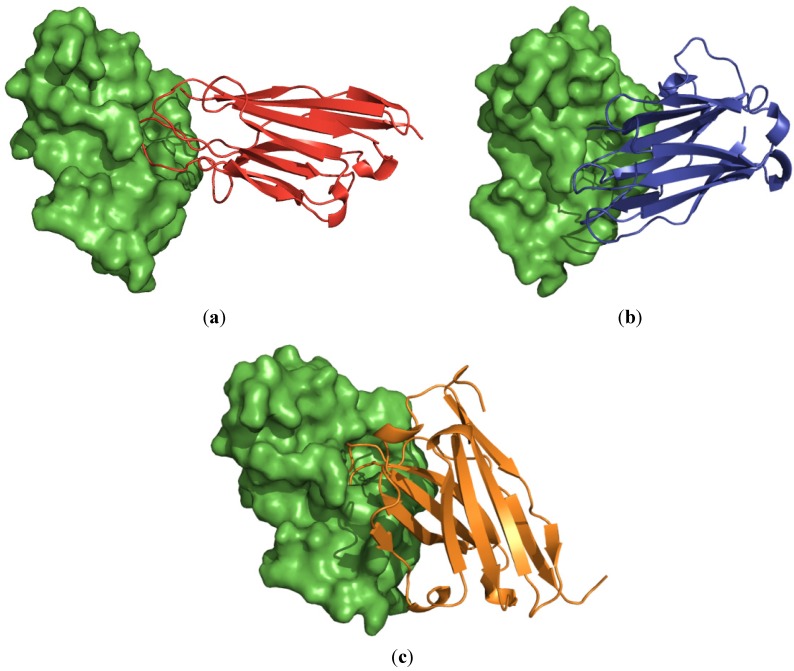
Hypothetical models showing binding sites of the antibodies (ribbons) V_H_H-P3-1 (red; panel **a**), V_H_H-P3-3 (blue; panel **b**) and VH P3-7 (orange; panel **c**) on the molecular surface of PLA_2_ (green surface model). All antibodies were found to cover the enzymatic groove surface areas of the modeled PLA_2_ and inserted the CDRs into the catalytic cleft.

The interface bindings of the antibodies and the PLA_2_ are shown in Figure 5. It was found that all antibodies occupied the PLA_2_ catalytic surface areas and protruded their CDRs into the PLA_2_ enzymatic groove.

## 4. Discussions

Phospholipase A2 (PLA_2_) of *Naja kaouthia* venom comprises both basic and acidic proteins [1,5]. The most toxic effect of the PLA_2_ is believed to be indirectly mediated by the catalytic active acidic PLA_2_ which consequently enhances cellular or subcellular (mitochondrial) membrane damage through phospholipid hydrolysis and generation of pharmacological mediators including platelet activating factor and eicosanoids causing hemolysis, hemorrhage, edema and loss of organ functions. Besides this, the PLA_2_ can cause prolonged blood coagulation, dermo-/myo-necrosis and cardiotoxicity [25]. In this study, attempts have been made to produce a humanized-single domain antibody (VH/V_H_H) that specifically interferes with the *N. kaouthia* PLA_2_ catalytic activity. 

Two isoforms of *N. kaouthia* PLA_2_, *i.e.*, P3 and P5, were isolated from the *N. kaouthia* venom [1]. These two proteins possessed the phosphatidylcholine hydrolytic activity as assessed in this study by using the commercialized sPLA2 assay kit. The P3 was more enzymatically active than the P5 on the same weight basis (0.47 *versus* 0.13 µmol/min/mL). When these two proteins were used separately as target antigens in phage bio-panning for selecting specific phage clones that displayed the antigen-bound humanized-camel VH/V_H_H on the surface and carried also the respective VH/V_H_H coding sequences in the phage genomes, it was found that only the P3 antigens could be selected for the desired phage clones which not only bind to the homologous P3 but also to the P5. None of the transformed *E. coli* clones transfected with the P5-derived phage clones produced VH/V_H_H that specifically bound to the P3/P5 PLA_2_. It is not known whether the difference in the antigenicity of the two proteins lies in their enzymatic activity, some amino acid differences or due to other factors. Moreover, P5-specific VH/V_H_H might be relatively rare in the antibody repertoire of the phage library. Similar results were obtained by using another antibody phage display library [26]. 

It should be noted that the P3-1 and P3-3 bound better to the heterologous P5 than to the homologous P3; the P3-7 bound equally well to both P3 and P5 in the indirect ELISA. Nevertheless, the antibodies of the three clones neutralized enzymatic activity of the homologous P3 enzyme (toxin: antibody 1:3) better than the heterologous P5 enzyme (toxin: antibody 1:5). The ambiguous results should be due to differences of the assay principles and the functions of the epitopes bound by the antibodies. Among the V_H_H-P3-1, V_H_H-P3-3 and VH-P3-7, the V_H_H-P3-3 showed the highest inhibitory activity to the P3 and P5 phospholipase activity, *i.e.*, 52% and 37%, respectively. The percent inhibition of the P3 and P5 PLA_2 _catalytic activity mediated by the horse immune serum to *N. kaouthia* venom (positive inhibition control of this study) were 35% and 45%, respectively. A previous study has demonstrated also that at dilution 1:500, the horse anti-*N. kaouthia* serum inhibited 16% of the snake PLA_2_ enzymatic activity [5]. Thus, the humanized-VH/V_H_H produced in this study has, more or less, comparable PLA_2_ enzyme inhibitory activity to the animal immune sera derived from two different sources. It is not known why the PLA2 specific antibodies in the preparations tested could not completely inhibit the PLA2 enzymatic activity. 

Molecular modeling and docking results suggested that the humanized-camel VH/V_H_H antibodies, bound to the surface areas around the enzymatic pocket and with CDR loops inserted into the PLA_2_ catalytic groove, would probably interfere with the substrate accessibility of the enzyme and thus accounted for the observed enzymatic activity inhibition. This speculation needs experimental validation. It is known that the surface areas of the PLA_2_ covered by the humanized-VH/V_H_H also involved in Ca^2+^ binding (residues Y27, G29, G31 and D48), active site (H47, D48, Y51 and D93), phospholipid binding (L2, F5, I9, W19, F21, A22, G31 and Y63) and anticoagulant activity (residues 54–66, *i.e*., AEKISRCWPYFKT) [27,28]. Thus by binding to the PLA_2_ target, the humanized-VH/V_H_H might exert not only the neutralization of the PLA_2 _enzyme, but also other PLA_2_ bio-toxic functions. Experiments are needed to verify this speculation. Moreover, the ability of the humanized-single domain antibodies (VH/V_H_H) in blocking the venom PLA_2_ mediated dermo-/myo-necrosis, hemorrhage and carditoxicity should also be explored. 

## 5. Conclusions

Humanized-camel single domain antibodies (VH/V_H_H) that cross neutralized enzymatic activity of different isoforms of *N. kaouthia* PLA_2_ were produced from an antibody phage display library. The PLA_2_ neutralizing activities of the VH/V_H_H were comparable to the horse immune serum against *N. kaouthia* holo-venom. Homology modeling and molecular docking revealed that the VH/V_H_H bound to the surface areas around the enzymatic pocket of the PLA_2 _and inserted the CDRS into the catalytic cleft.

## Supplementary Files

Supplementary File 1: PDF-Document (PDF, 621 KB)
