# Supplementary material for: Humanized-Single Domain Antibodies (VH/VHH) that Bound Specifically to Naja kaouthia Phospholipase A2 and Neutralized the Enzymatic Activity
Source: Toxins (Basel). 2012 Jul 19;4(7):554–67. doi: 10.3390/toxins4070554 (PMC3407892; doi:10.3390/toxins4070554)

Supplementary Information

Supplemental Figure 1. SDS-PAGE-separated P3 (lane 1) and P5 (lane 2) stained with Coommassie Brilliant Blue G-250. Lane M, Pre-stained protein marker. Numbers at the left are protein molecular masses in kDa.

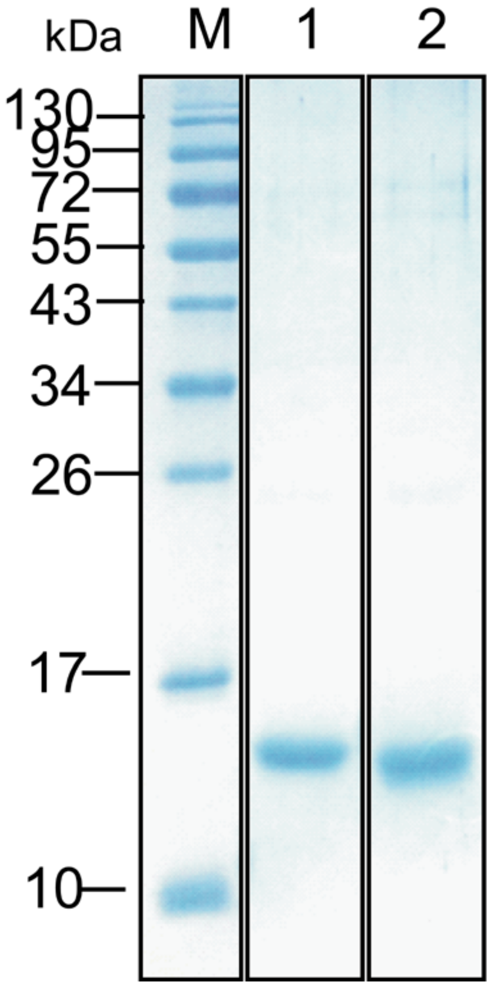

Supplemental Figure 2. Mass spectra of the protein in P3 (A) and P5 (B) fractions derived from ion exchange chromatographic separation of the holo-venom of N. kaouthia. The P3 and P3 were identified by LC-MS/MS as phospholipase A2.

A

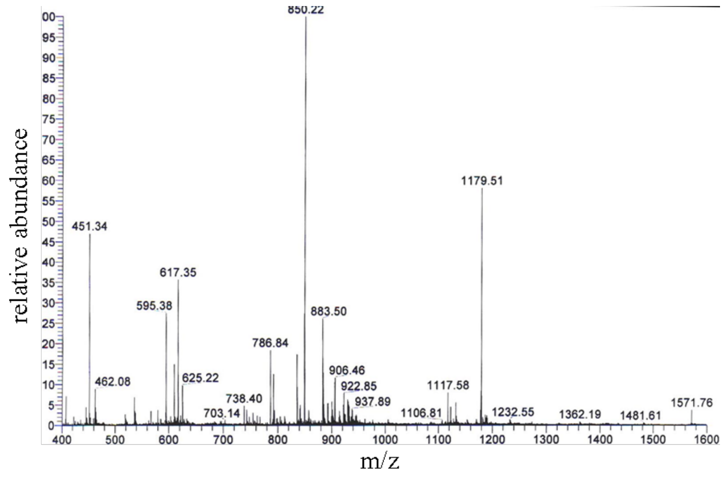

B

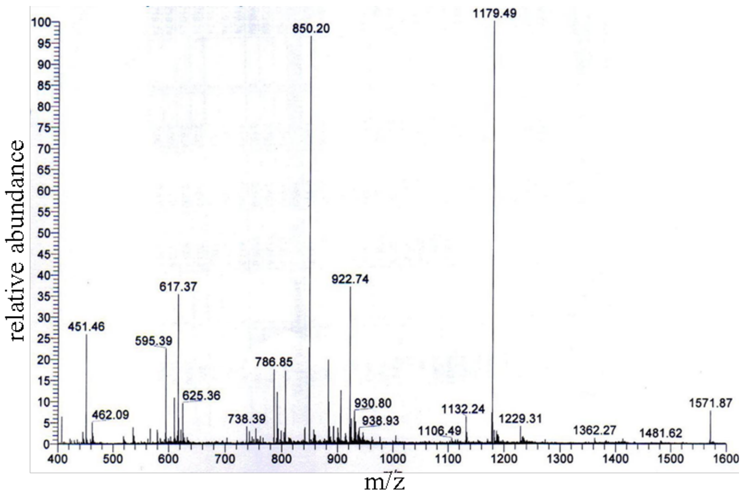

Supplemental Figure 3. Patterns of humanized-VH/V_HH in SDS-PAGE separated lysates of representative *vh/v_hh*-phagemid transformed *E. coli* clones. Lane M, Standard protein markers. Lanes 1-7, SDS-PAGE separated lysates of 7 transformed *E. coli* clones; the VH/V_HH appeared in most lanes as a protein doublet which the lower band was mature VH/V_HH while the upper band was the protein with signal peptide. The lowest band in lane 3 is degraded product of the principal protein. Lane 8, SDS-PAGE separated lysate of normal *E. coli* (negative VH/V_HH control). Numbers at the left are protein sizes in kDa.

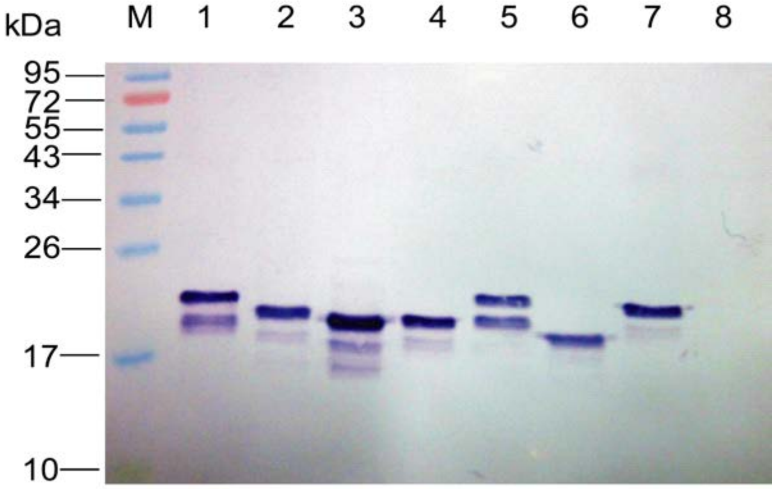

Supplemental Figure 4. Ramachandran plots of modeled structures of VH/V_HH (A) and PLA_2 (B).

A

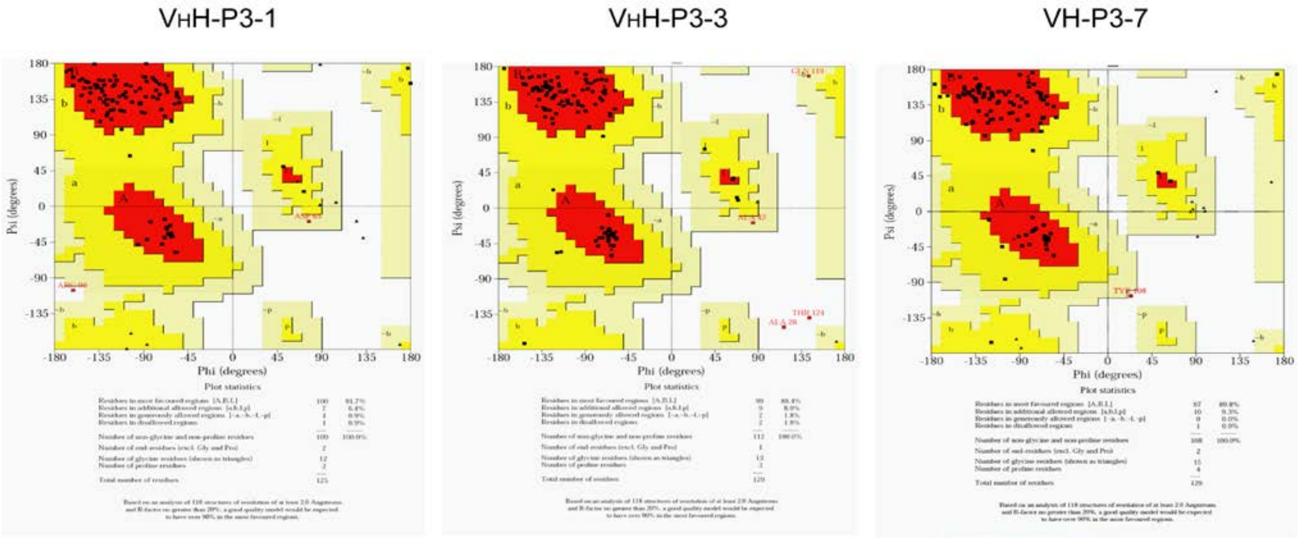

B

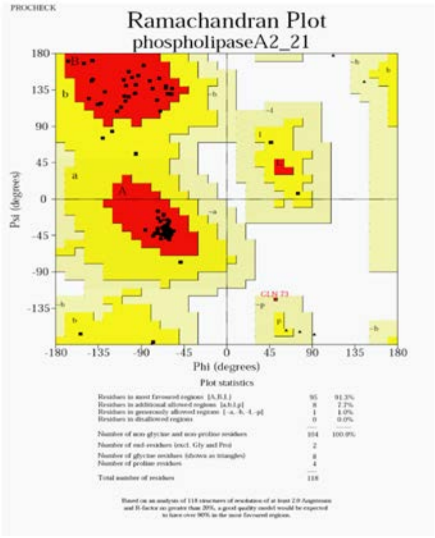

Supplement: Supplementary File 1: — PDF-Document (PDF, 621 KB) [file toxins-04-00554-s001.pdf]
